# User’s Search for Information: A Multi-Language Cross-Sectional Assessment of Websites about Healthcare-Associated Infections

**DOI:** 10.2478/jccm-2024-0011

**Published:** 2024-01-30

**Authors:** Valentin Nădășan, Dalma Kasza, Konrád-Ottó Kiss, David Maior, Marius Mărușteri

**Affiliations:** Hygiene Department, George Emil Palade University of Medicine, Pharmacy, Science, and Technology of Targu Mures, Romania; Neurology Clinic II, County Emergency Clinical Hospital of Targu Mures, Romania; Anesthesiology and Intensive Care Clinic, County Emergency Clinical Hospital of Targu Mures, Romania; Trauma and Ortopedics Clinic, County Emergency Clinical Hospital of Targu Mures, Romania; Medical Informatics and Biostatistics Department, George Emil Palade University of Medicine, Pharmacy, Science, and Technology of Targu Mures, Romania

**Keywords:** infodemiology, health-related information, internet, quality assessment, consumer health informatics, misinformation, nosocomial infections

## Abstract

**Introduction:**

Healthcare-associated infections have a significant impact on public health, and many patients and their next-of-kin are seeking information on the internet. The study aimed to assess the quality of online written content about healthcare-associated infections available in English, Romanian, and Hungarian languages.

**Materials and methods:**

The study sample included 75 websites, 25 for each language subgroup. The assessment involved examining the general characteristics, adherence to established credibility criteria, and the completeness and accuracy of informational content. The evaluation was conducted using a topic-specific, evidence-based benchmark. Two evaluators independently graded completeness and accuracy; scores were recorded on a scale from 0 to 10. A comparative analysis of websites was performed, considering pertinent characteristics, and potential factors influencing information quality were subjected to testing. The statistical significance was set at 0.05.

**Results:**

For the overall study sample, the average credibility, completeness, and accuracy scores were 5.1 (SD 1.7), 2.4 (SD 1.5), and 5.9 (SD 1.0), respectively. Pairwise comparison tests revealed that English websites rated significantly higher than Romanian and Hungarian websites on all three quality measures (P<0.05). Website specialization, ownership, and main goal were not associated with credibility or content ratings. However, conventional medicine websites consistently scored higher than alternative medicine and other websites across all three information quality measures (P<0.05). Credibility scores were positively but weakly correlated with completeness (rho=0.273; P=0.0176) and accuracy scores (rho=0.365; P=0.0016).

**Conclusions:**

The overall quality ratings of information about healthcare-associated infections on English, Romanian, and Hungarian websites ranged from intermediate to low. The description of information regarding the symptoms and prevention of healthcare-associated infections was notably unsatisfactory. The study identified website characteristics possibly associated with higher-quality online sources about healthcare-associated infections, but additional research is needed to establish robust evidence.

## Introduction

As early as the 19th century, Semmelweis’s ground-breaking work shed light on the potential for disease transmission within healthcare settings. These early observations set the stage for the recognition and understanding of nosocomial or hospital-associated infections, emphasizing the critical need for rigorous infection control measures to safeguard both patients and healthcare providers [1,2].

Healthcare-associated infection refers to “An infection occurring in a patient during the process of care in a hospital or other health care facility, which was not present or incubating at the time of admission” [3]. More precisely, the symptoms must not be present on the day of admission and need to appear after 48th hours after the admission, in the first three days after discharge, 30 days of an operation or after a year of an implant [4]. Nosocomial infection and healthcare-associated infection (HAI) are terms that are often used interchangeably and refer to infections that are acquired in a healthcare setting. While the population at highest risk of HAI includes individuals in intensive care units, burns departments, organ transplant centers, and neonate wards, these infections can occur not only in hospitals and clinics but also in nursing homes, rehabilitation facilities, and other healthcare environments. Compared to nosocomial infections, HAI is a broader concept emphasizing the wider scope of infections that can occur within the healthcare system beyond just hospital settings [5,6,7].

According to the latest WHO global report on infection prevention and control, among patients in acute-care hospitals, approximately seven individuals in high-income countries and 15 individuals in low- and middle-income countries will experience at least one HAI out of every 100 patients. Notably, the incidence in low- and middle-income countries is reported to be 2 to 20 times higher than in high-income countries, particularly among neonates [8]. A recent systematic review and meta-analysis including 400 studies estimated that the worldwide rate of universal HAIs is increasing annually with 0.06 [9]. HAIs stand out not only as the most prominent patient safety issue but also as the most expensive hospital-related adverse event. For example, in the United States, hospitals face an annual direct cost of HAIs ranging from $28 billion to $45 billion [8,10].

Patients in intensive care units (ICU) have a considerably higher risk of HAIs and subsequent severe complications and death. Data reported by the World Health Organization’s World Alliance for Patient Safety program show that in ICU, around 30 out of every 100 patients experience HAIs, and the mortality linked to these infections can increase up to 44%. The risk of HAIs in ICU is higher because more patients already have severe and multiple health problems, weakened immune systems, and are older. Additionally, the increased use of invasive medical devices, crowded conditions, and emergencies make it challenging to control and prevent infections effectively [3].

The European Centre for Disease Prevention and Control estimates that each year, 3.1–4.6 million individuals contract HAIs, and over 90,000 people lose their lives in the European Union countries, Iceland, Norway, and the United Kingdom [11,12]. The recorded rate of HAIs in public hospitals in Romania is less than 1%. Intensive care units reported the highest occurrence of HAIs, with the most prevalent types being bronchopneumonia, enterocolitis, sepsis, surgical wound infections, and urinary tract infections. However, there are concerns that the actual number might be higher because the medical staff may not be fully trained, and HAI diagnoses may be low. While there are not many studies on HAI in Romania, the existing ones hint at a much higher prevalence than officially reported [13,14]. Based on the ECDC point prevalence survey for 2016–2017, the reported rate of HAIs in long-term care facilities in Hungary was 0.9% [15].

Globally, the most frequent locations often include the urinary tract, respiratory system, digestive tract, surgical wound, and skin [16]. The most prevalent infection types encompass central line-associated bloodstream infections, catheter-associated urinary tract infections, surgical site infections, and pneumonia linked to ventilator use [6].

Studies show that HAIs are associated with several risk factors. Sociodemographic characteristics, such as age above 60 years and male sex, were found to be significant risk factors. Invasive procedures, chronic diseases, coma, and immunosuppression were also linked to increased HAI risk. Additional factors included prolonged bedridden status, healthcare-related elements like chemotherapy, hemodialysis, hormone therapy, and antibiotic usage [17,18,19,20].

While healthcare professionals and institutions play a crucial role in infection prevention, patients can impact the risk of infection through their actions. Important factors related to the patient’s behavior before and during hospitalization are: poor hygiene practices (handwashing, oral hygiene) [21,22]; noncompliance to prescribed treatments, especially antibiotics or not following wound care instructions [23,24]; nonadherence to personal protective measures (facial masks, isolation protocols) [25]; neglecting vaccination, especially against vaccine-preventable diseases, can increase the likelihood of contracting and spreading certain infections [26]; patients and visitors not observing guidelines for responsible visitation, especially during outbreaks [27].

Involving patients and their families in efforts to prevent HAIs can be beneficial. Patient empowerment through providing knowledge and tools to actively participate in preventing infections is crucial because patients often lack awareness of the risks of infections related to medical and surgical procedures. By educating patients and their families, there is a potential to enhance their understanding and engagement in infection prevention, ultimately reducing the occurrence of HAIs [28].

Surveys show most of the general public is aware of HAIs, multiply resistant bacteria, and the importance of hand hygiene in the prevention of infections, and consider these parameters when choosing a hospital, but data are indicating that many patients do not receive explanations about the risks before admission, and most of them are dissatisfied with the information they were provided [29,30,31]. Hardly surprising, data show a significant proportion of next of kin of patients admitted to the intensive care units using very frequently the internet as a source of information [32].

Considering the significant impact of HAIs on public health, the widespread reliance on the internet as a key source of information on a large array of conditions, and the distressing and misleading potential of misinformation on patient and caregiver health-seeking behavior [33,34,35,36,37], this study aimed to assess the quality of online content about HAIs available in English, Romanian, and Hungarian languages. Additionally, the study explored the potential correlates of high-quality websites providing information about HAIs.

## Methods

This research is part of a comprehensive medical information hygiene project that aims to assess the quality of information about the most prevalent diseases in several languages. To date, the project has covered 43 medical topics, including several that are closely related to first aid, emergency medicine, and critical care, such as stroke, myocardial infarction, coronary angioplasty, first aid in case of burn, first aid in case of choking and applied a standard methodology as described in previously published works [38,39,40,41].

### Sample selection

The sample of this cross-sectional observational study included 75 websites about HAIs designed for the general population in English, Romanian, and Hungarian (25 websites for each language).

To simulate searches performed by general users, the query terms were identified based on popularity statistics retrieved from Google Trends, a tool that analyses the frequency of query terms in Google searches across various regions and languages. The top terms were “nosocomial infection” for English, “infecție nosocomială” for Romanian, and “kórházi fertőzés” for Hungarian.

Next, the chosen query terms were employed on the Google Search engine to find the topic-relevant websites. To narrow down the process to a particular language, the localized and language-specific versions of the engine were employed (“www.google.com” for English, “www.google.ro” for Romanian, and “www.google.hu” for Hungarian). For each language, the first 100 results were saved by default. The search and selection procedures were performed between February and December 2022.

Candidate websites listed on the Google results page were screened based on the following inclusion criteria: the main topic was HAIs; text content in the specific language; minimum word count of 300; website addressing the general population. Additionally, the following exclusion criteria were applied: websites consisting exclusively of audio or video content; websites that presented the topic of interest in the form of brief news and comments on forums and social networks; websites allowing access after registration or payment; infected and inaccessible pages; and sponsored websites [38,39].

### Assessment procedures

The websites’ general characteristics (specialty, ownership, main goal, website type, and medical paradigm) and their compliance with 12 credibility criteria for health-related websites were assessed by one author using a predesigned assessment form. The credibility criteria were derived from three internationally recognized authorities in the field of consumer health informatics and infodemiology: eEurope 2002 quality criteria for health-related websites [42], Health on the Net (HON) code of conduct [43], and JAMA benchmark for online medical information [44]. Each specific credibility criterion was coded as either 1 for compliance or 0 for noncompliance. Subsequently, a scale variable from 0 to 10 was calculated by summing the number of individual credibility criteria with which websites complied. This scale variable served as an indicator of overall compliance with credibility requirements [38,39].

The second stage of the process investigated the topic-specific informational content of the selected web pages considering two basic dimensions of quality, namely, completeness and accuracy. This step was performed by two authors independently, each adhering to a detailed list of instructions. Completeness and accuracy were measured against a predefined, evidence-based quality benchmark, critically reviewed by two specialists (one epidemiologist and one hygienist). The benchmark comprised 50 items covering knowledge about HAIs structured into the following sections: definition, epidemiology, etiology, risk factors, types and localization of HAIs, evolution and complications, treatment, and prevention.

Considering completeness, the presence of an item in the text was coded with 1, and the absence of the idem was coded with 0. The sum of the addressed items on a website yielded the overall completeness score of the respective website. Considering accuracy, each item on the website was assessed using a three-level scale: correct items were coded with 2, mostly correct items were coded with 1, and mostly incorrect items were coded 0. The cumulative points awarded to a site resulted in the overall accuracy score of the respective site. The initial simple summative scores were converted to scales ranging from 0 to 10 to facilitate site comparison [38,39].

Four dimensional subscores were also computed to assess the following areas of information: A. General information about HAIs (definition and epidemiology); B. The causes and risk factors of HAIs; C. The clinical characteristics of the most common HAIs; D. Treatment and prevention of HAIs.

### Statistical Analysis

The quality scores were presented as mean values and standard deviations (SD). Inter-rater concordance of completeness and accuracy assessments were checked using Cohen’s kappa test. Kappa coefficients of 0.8 or higher were considered very good. Websites with kappa values less than 0.8 underwent a second joint assessment to settle the differences by consensus.

The Kolmogorov-Smirnov test was used to check the normality of the data. Differences regarding credibility, completeness, and accuracy between two independent groups were analyzed with the Mann-Witney test while differences between three or more independent groups were examined with Kruskal-Wallis test. The Spearman correlation test was used to check if the websites’ compliance with the credibility criteria and their Google ranks are potential correlates of high completeness and accuracy. The statistical analyses were conducted using IBM SPSS statistical package v.22. The significance level was set at 0.05.

## Results

The distribution of the studied websites according to their general characteristics by language is shown in Table 1.

**Table 1.** The number (n) and relative frequencies (%) of the websites presenting information about healthcare-associated infections by general characteristics and language

**General Characteristics**	**Language of the websites**			

	**English (N=25)**	**Romanian (N=25)**	**Hungarian (N=25)**	**Total (N=75)**
**Specialization**				
Single medical specialty, n (%)	8 (32.0)	6 (24.0)	7 (28.0)	21 (28.0)
Multiple medical specialties, n (%)	17 (68.0)	19 (76.0)	18 (72.0)	54 (72.0)

**Website ownership**				
Foundation, NGO, n (%)	9 (36.0)	4 (16.0)	3 (12.0)	16 (21.0)
Private or state health care provider, n (%)	2 (8.0)	3 (12.0)	4 (16.0)	9 (12.0)
Commercial company, n (%)	7 (28.0)	16 (64.0)	13 (52.0)	36 (48.0)
Medical product manufacturer/distributor, n (%)	3 (12.0)	2 (8.0)	2 (8.0)	7 (9.3)
Educational or research institution, n (%)	4 (16)	0	1 (4)	5 (6.7)
Unidentifiable, n (%)	0 (0.0)	0 (0.0)	2 (8.0)	2 (2.7)

**Main purpose**				
Educational, n (%)	18 (72.0)	17 (68.0)	19 (76.0)	54 (72.0)
Commercial, n (%)	6 (24.0)	6 (24.0)	5 (20.0)	17 (22.7)
Socialization or support, n (%)	1 (4.0)	2 (8.0)	1 (4.0)	4 (5.3)

**Website format**				
Thematic, n (%)	6 (24.0)	2 (8.0)	4 (16.0)	12 (16.0)
General portal, n (%)	0 (0.0)	0 (0.0)	2 (8.0)	2 (2.7)
Medical portal, n (%)	7 (28.0)	1 (4.0)	4 (16.0)	12 (16.0)
Electronic publication, n (%)	2 (12.0)	5 (20.0)	1 (4.0)	8 (10.7)
Company presentation page, n (%)	3 (12.0)	7 (28.0)	3 (12.0)	13 (17.0)
Web-based shop, n (%)	2 (8.0)	9 (36.0)	8 (32.0)	19 (25.3)
Blog or personal page, n (%)	1 (4.0)	0 (0.0)	0 (0.0)	1 (1.3)
Other, n (%)	4 (16.0)	1 (4.0)	3 (12.0)	8 (10.7)

**Medical paradigm**				
Conventional medicine, n (%)	25 (100.0)	4 (16.0)	3 (12.0)	32 (42.7)
Alternative medicine, n (%)	0 (0.0)	0 (0.0)	2 (8.0)	2 (2.7)
Mixed approach, n (%)	0 (0.0)	3 (12.0)	3 (12.0)	6 (8.0)
Unidentifiable, n (%)	0 (0.0)	18 (72.0)	17 (68.0)	35 (46.7)

**Fig. 1.**
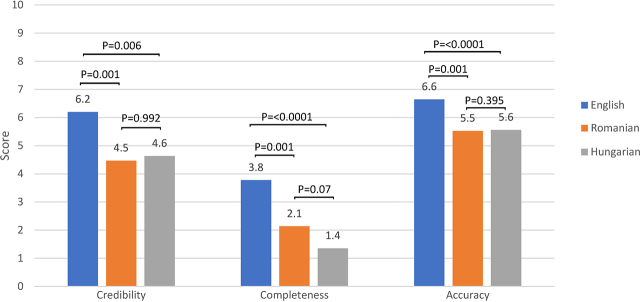
The mean credibility, completeness, and accuracy scores of information about healthcare-associated infections and the Mann-Whitney P-values of the pairwise comparison tests between the English, Romanian, and Hungarian language websites

The means and SDs of the credibility, completeness, and accuracy scores for the whole study sample were 5.1 (SD 1.7), 2.4 (SD 1.5), and 5.9 (SD 1.0), respectively. The Kruskal-Wallis test results showed a statistically significant difference between language subsamples concerning credibility (P=0.0021), completeness (P<0.0001), and accuracy (P=0.0002). The mean information quality scores of the three language subsamples and the results of the pairwise comparison tests are presented in Figure 1.

The means and SDs of the dimensional completeness scores for the whole sample (all three languages considered together) were: A. general information: 3.9 (SD 3.0); B. information about causes and risk factors: 3.7 (SD 3.3); C. information about symptoms: 1.6 (SD 1.7); D. information about treatment and prevention: 2.3 (SD 1.8). The means and SDs of the dimensional accuracy scores for the whole sample were: general information: 6.1 (SD 1.6); information about causes and risk factors: 6.2 (SD 1.5); information about symptoms: 6.3 (SD 1.5); information about treatment and prevention: 6.0 (SD 1.2).

**Fig. 2.**
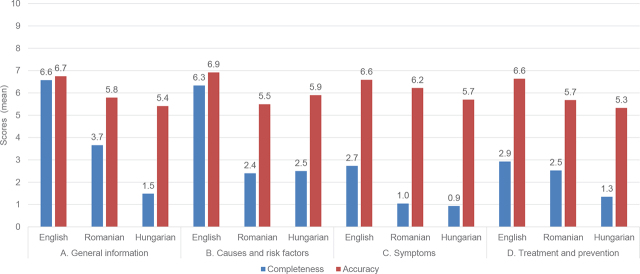
The mean dimensional completeness and accuracy scores by language subsamples: A. General information; B. Causes and risk factors; C. Symptoms; D. Treatment and prevention

The mean values of the dimensional completeness and accuracy scores by language subsample are presented in Figure 2.

The differences between the websites’ credibility, completeness, and accuracy scores depending on their general characteristics are presented in Table 2.

**Table 2.** The mean values and SDs of the credibility, completeness, and accuracy scores by the websites’ general characteristics

**General characteristics**		**Credibility, mean (SD)**	**P-value**	**Completeness, mean (SD)**	**P-value**	**Accuracy, mean (SD)**	**P-value**
Topic	Single	4.7 (1.8)	0.21^a^	2.4 (1.7)	0.920^a^	5.8 (0.8)	0.423^a^
	Multiple	5.3 (1.9)		2.4 (1.9)		6.0 (1.3)	

Owner	NGO	5.9 (1.5)	0.1526^b^	2.9 (1.7)	0.6^b^	6.3 (0.9)	0.1573^b^
	Health service provider	4.9 (2.2)		2.1 (1.5)		5.9 (1.0)	
	Commercial company	4.8 (1.9)		2.3 (1.9)		5.6 (1.2)	
	Other	5.5 (1.7)		2.6 (2.4)		6.5 (1.4)	

Goal	Educational or networking	5.3 (2.0)	0.0533^a^	2.5 (1.9)	0.6033^a^	6.0 (1.3)	0.1487^a^
	Commercial	4.3 (1.3)		2.2 (1.6)		5.7 (0.8)	

Website type	Topic-based content	5.9 (1.6)	0.0008^b^	2.5 (2.1)	0.0586^b^	6.7 (1.1)	0.0113^b^
	Medical portal	6.7 (1.4)		3.1 (2.0)		5.9 (0.8)	
	Company promotion	3.8 (1.3)		2.5 (1.7)		5.7 (0.7)	
	Online shop	4.4 (1.4)		1.4 (1.4)		5.3 (1.5)	
	Other	5.2 (2.2)		2.9 (1.7)		6.3 (1.0)	

Medical paradigm	Conventional	6.0 (1.8)	<0.0001^a^	3.3 (1.8)	<0.0001^a^	6.4 (1.1)	0.005^a^
	Alternative and other	4.4 (1.7)		1.7 (1.5)		5.6 (1.1)	

a: Mann-Whitney test;

b: Kruskal-Wallis test

The websites’ compliance with the 12 individual credibility criteria is presented in Table 3.

**Table 3.** Prevalence of websites about healthcare-associated infections complying with the individual credibility criteria

**Criteria**	**English subsample (%)**	**Romanian subsample (%)**	**Hungarian subsample (%)**	**Whole sample (%)**
Owner name and address	100	84	100	95
Mission statement	100	96	68	88
Consultation disclaimer	44	4	20	23
Disclosure of funding	52	16	40	36
Disclosure of commercial interest	80	8	4	31
Confidentiality statement	92	60	60	71
Providing contact mechanism	96	92	96	95
Authors’ credentials	44	36	32	37
Referencing sources	40	16	16	24
Display publication date	24	88	84	65
Displaying the date of last update	40	24	24	29
Quality procedure statement	32	12	12	19

The results of testing the correlations between the credibility scores and Google ranks, on the one hand, and the content quality scores, on the other hand, for HAI-related websites in all three languages are reported in Table 4.

**Table 4.** Correlations between the credibility score and Google ranks versus completeness and accuracy scores of websites presenting information about healthcare-associated infections in English, Romanian, and Hungarian language

**Independent variable**	**Dependent variable**	**Spearman rho**	**P-value**
Credibility score	Completeness score	0.273	0.0176
	Accuracy score	0.365	0.0016

Google rank	Completeness score	−0.112	0.3389
	Accuracy score	−0.168	0.158

## Discussion

Overall, the quality of online information about HAIs in English, Romanian, and Hungarian ranged from intermediate to low. All three language subsamples’ credibility and accuracy scores were in the middle range of the scale (between 4 and 7), while the completeness scores were low or very low (between 1 and 4). The absence of website ratings above eight on completeness and accuracy scales also indicated the lack of high-quality websites. These observations suggest that internet users may have difficulty finding complete and accurate information about HAIs on a single website. Obtaining comprehensive and correct information on this topic would require accessing several websites in a complementary manner.

Data on the quality of information about conditions needing critical care are extremely scarce, but plenty of studies are assessing other medical topics. Although rigorous comparisons are not applicable because of methodological differences, most of the authors conclude the quality of health-related information on the internet is low or acceptable, at most [45,46,47].

Statistically significant differences were detected between the three language subsamples regarding every quality score. The pairwise comparisons showed that the English websites were superior on all three quality indicators compared to the Romanian and Hungarian websites, and the differences were statistically significant. However, the practical significance of these differences is questionable, considering that the English website’s quality scores were only 1.0 to 2.3 points above the Romanian and Hungarian website’s quality scores. No statistically significant differences were detected between the Romanian and Hungarian websites.

While these observations suggest that internet users who understand English are more likely to access better sources of information about HAIs, the advantage does not seem impressive when looking at the completeness and accuracy ratings (Figure 1). These observations are supported by the findings of a few other analyses conducting multi-language comparisons, but more studies are needed to draw a general conclusion [39,48].

The completeness and accuracy dimensional scores follow the pattern of overall quality scores with low completeness and relatively higher accuracy. A closer look at the variations across the four dimensions shows that information about the clinical manifestations and prevention of HAIs is especially lacking, with ratings as low as 1.6 and 2.3, respectively. These observations could help health professionals and institutions involved in educational efforts to develop informative websites to cover the underrepresented notions about HAIs. These deficiencies are more striking on the Romanian and Hungarian websites than English ones.

There were no statistically significant differences regarding the completeness and accuracy scores of websites classified according to specialization, ownership, and main goal. However, the analysis of website types suggested that medical portals may have slightly higher credibility scores, and topic-based websites may have a slightly higher accuracy score than other websites. It is also worth noting that websites with a conventional medicine approach had consistently higher scores than alternative medicine and other websites on all three information quality measures. The differences were statistically significant (credibility: P<0.0001; completeness: P<0.0001; and accuracy: P=0.005), but the amplitude of differences would not be practically useful if the paradigm of websites would be used as a predictor of information quality (Table 2). Other authors reported higher quality of information on institutional websites and lower content quality on alternative medicine websites [49,50].

Overall, the most frequently observed credibility criteria were displaying the owner’s name and address (95% of the websites), providing a contact mechanism for users (95% of the websites), disclosing the mission statement (88% of the websites), and providing a confidentiality statement (71% of the websites). The study identified three areas of concern regarding those credibility requirements that experts believe are the most stringent for the medical internet: (a) only 23% of the websites displayed a consultation disclaimer (warning the users that the information provided online should not replace the personal consultation given by doctors); (b) less than 40% of the examined webpages mentioned the name and credentials of the content’s author, suggesting the many of the articles were written or compiled by individuals without formal medical training; (c) only 29% of the websites in the studied sample provided the date of the last update of the content. This observation raises serious concerns because the timeliness of information is essential in medicine.

The websites’ credibility scores significantly correlated with completeness and accuracy scores. While the correlation was positive, as expected, the intensity of the correlations was weak. This suggests that compliance with credibility requirements may not be a reliable predictor of content quality. Reports in the literature about this correlation show inconsistent findings, with some suggesting weak positive correlation and others no correlation [39,51,52]. In the present study, the Google ranks of the websites, probably the most user-convenient indicator of website quality, were not correlated with the completeness or accuracy of the studied sources of information about HAIs. In this respect, there are also discrepancies between data reported in the literature, and more studies are warranted to provide solid evidence [49,53].

The results of our study have important implications for risk communication regarding HAIs and the practice of infection prevention and control (IPC) programs. Misinformation, especially when growing into infodemics, can dramatically impact the healthcare system at large and ED and ICU in particular. Experts suggest that infodemics can diminish the public’s confidence in the health system and impede access to evidence-based health services by causing delays in the provision of treatment to patients. All these can lead to an increased number of critically ill cases, augmentation of ICU burden, and finally, excess mortality. Therefore, situational analysis conducted before IPC educational program development should incorporate quantitative and qualitative assessments to identify the prevalent HAIs-related misinformation, the most influential sources disseminating such content, and its potential impact on infection prevention and control. Legislators and local health facility administrators in charge of IPC programs should understand the infodemiological mechanisms and, thus, address rationally the vulnerabilities attributable to lack of awareness and exposure to misinformation regarding HAIs through proactive, tailored risk communication messages [54,55].

### Strengths and limitations

To the best of our knowledge, this study is the first investigation on the quality of information about HAIs in multiple languages on sources intended for the general population. The only article that aimed to evaluate the quality of infectious disease hospital websites in Poland investigated only website accessibility, performance, content readability, and graphical appeal, did not focus on HAIs, and did not address the issue of completeness and scientific accuracy of information [55].

Thus, the insights gained from our study could provide valuable guidance to experts and institutions in charge of public education regarding HAIs prevention through dissemination of complete and scientifically accurate information directly to the population and, indirectly via mass-media channels. Just to underline the importance of the above conclusion, it is worth mentioning that, in an opinion paper, researchers from Spain and the USA expressed the perception that HAIs receive minimal or no coverage in daily news, particularly on television, and urged the responsible actors for remediation of the issue [56].

The study has certain limitations associated with the inherent characteristics of online research. Internet users may employ various search engines and perform queries with different keywords, resulting in varied search outcomes. Additionally, the dynamic nature of the online environment makes it challenging to replicate the study’s results precisely. Furthermore, the popularity of the AI-driven generative large language models such as ChatGPT (Open AI), Bard AI (Google), and Llama (Meta) is expected to considerably reshape the information-seeking behavior among internet users in the near future. Nonetheless, by emulating a popular search strategy employed by lay internet users, our findings likely mirror relevant user experiences to date.

Despite these limitations, the results of this investigation may provide valuable insights for the general users, patients, next-of-kin, and professionals involved in designing and planning internet, classical media, and social media-based educational campaigns on HAI awareness and prevention.

## Conclusions

The quality of information about HAIs, as investigated on English, Romanian, and Hungarian websites, was intermediate or low. Information about the symptoms and prevention of HAIs was particularly unsatisfactory. Some website characteristics, medical paradigm, and compliance to credibility criteria may indicate higher quality online sources about HAIs, but further research is needed to produce solid, practicable evidence.
